# Supplementary material for: Resveratrol-Induced White Adipose Tissue Browning in Obese Mice by Remodeling Fecal Microbiota
Source: Molecules. 2018 Dec 18;23(12):3356. doi: 10.3390/molecules23123356 (PMC6321286; doi:10.3390/molecules23123356)
Supplement: Supplementary file 1 [file molecules-23-03356-s001.pdf]

molecules

MDPI

*Supplementary*

# Resveratrol Induced WAT Browning in Obese Mice by Remodeling Fecal Microbiota

**Weiyao Liao [1,2,†], Xiaohan Yin [1,2,†], Qingrong Li [1,2], Hongmin Zhang [1,2], Zihui Liu [1,2], Xinjie Zheng [1,2], Lin Zheng [1,2] and Xiang Feng [1,2,*]**

[1] Department of Nutrition, School of Public Health, Sun Yat-sen University; Guangzhou 510080, China; liaowy6@mail2.sysu.edu.cn (W.L.); yinxh5@mail2.sysu.edu.cn (X.Y.); liqr9@mail2.sysu.edu.cn (Q.L.); zhanghm7@mail2.sysu.edu.cn (H.Z.); liuzihui@mail2.sysu.edu.cn (Z.L.); zhengxj27@mail2.sysu.edu.cn (X.Z.); zhenglin@mail.sysu.edu.cn (L.Z.); fengx@mail.sysu.edu.cn (X.F.)

[2] Guangdong Provincial Key Laboratory of Food, Nutrition and Health, Guangzhou 510080, China

* Correspondence: fengx@mail.sysu.edu.cn; Tel: +86-20-87332571

† Both authors contributed equally to this work.

Academic Editor: Min-Hsiung Pan; Filomena Conforti

Received: 22 November 2018; Accepted: 16 December 2018; Published: date

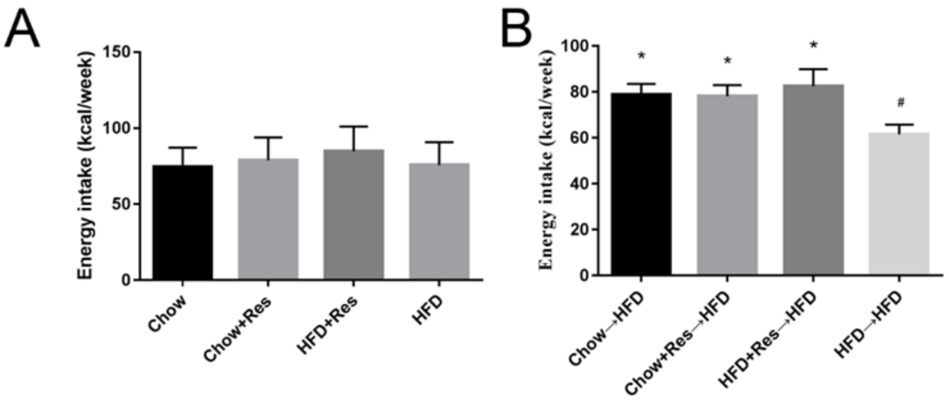


**Figure S1.** Fecal microbial transplants from donor to recipient mice.

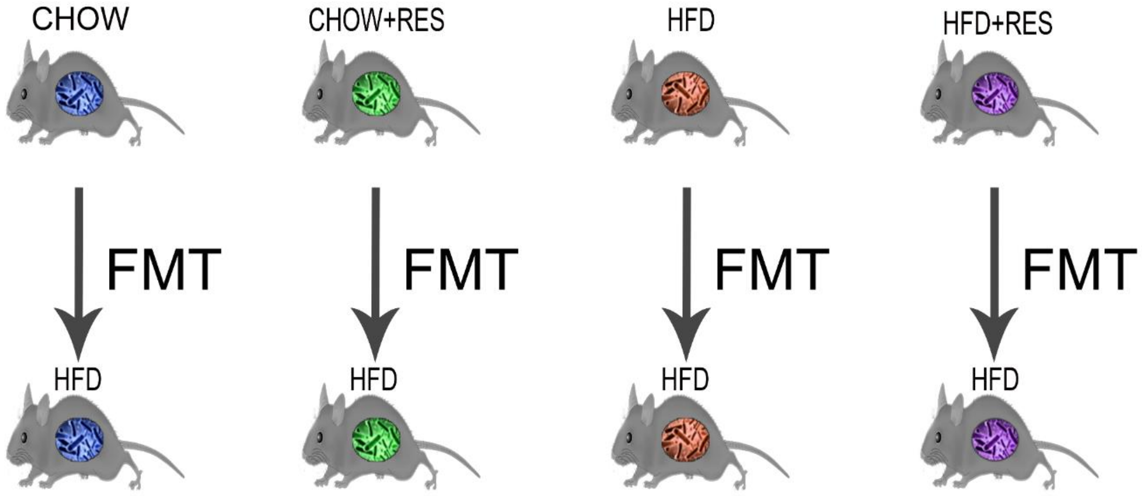


**Figure S2.** Energy intake by donor and recipient groups. Energy intake in donor groups (a) and recipient groups (b) was monitored (*n* = eight or seven for each group). Energy intake was determined based on caloric intake from consumed food. *$p < 0.05$ compared with HFD-CT or HFD-HFD.
